# Small colony variants and cefiderocol resistance in clinical *Escherichia coli*: an *in vitro* mechanistic study

**DOI:** 10.3389/fmicb.2026.1761368

**Published:** 2026-05-26

**Authors:** Muhammad Ali Syed, Danni Pu, Rongrong Song, Jiankang Zhao, Bin Cao

**Affiliations:** 1 State Key Laboratory of Respiratory Health and Multimorbidity, Department of Pulmonary and Critical Care Medicine, Center of Respiratory Medicine, National Center for Respiratory Medicine, National Clinical Research Center for Respiratory Diseases, Institute of Respiratory Medicine, China-Japan Friendship Hospital, Chinese Academy of Medical Sciences, Beijing, China; 2 Department of Microbiology, The University of Haripur, Haripur, Pakistan; 3 Graduate School of Peking Union Medical College, Chinese Academy of Medical Sciences, Peking Union Medical College, Beijing, China; 4 Department of Pulmonary and Critical Care Medicine, Capital Medical University, Beijing, China

**Keywords:** antibiotic resistance, cefiderocol, *Escherichia coli*, small colony variants, whole genome sequencing

## Abstract

**Background:**

The emergence of cefiderocol resistance in *Escherichia coli* poses a threat to this last line antibiotic against carbapenem-resistant infections. This study aimed to induce and characterize the mechanisms of cefiderocol resistance *in vitro*.

**Methods:**

Cefiderocol resistance was induced in four clinical *E. coli* strains (carrying *bla*_NDM-5_, *bla*_KPC-2_, *bla*_OXA-48_, *bla*_IMP-4_) and ATCC 25922 via serial passaging in increasing drug concentrations. Resistant strains were subjected to whole-genome sequencing (WGS) and RNA sequencing (RNA-Seq) to identify genetic determinants. Phenotypic changes were assessed through growth kinetics, biofilm formation assays, and transmission electron microscopy (TEM).

**Results:**

Resistance developed in all strains within 4–59 days. Small-colony-variant (SCV) emerged in all strains except K66460L (carrying *bla*_NDM-5_), which developed resistance within only 4 days. The resistant strains showed reduced biofilm production and impaired growth kinetics. WGS revealed mutations in genes related to iron transport, membrane function, and metabolic pathways. RNA-Seq confirmed altered expression of iron transport genes. TEM showed that resistant strains had an increased cell wall diameter. In addition, results of our study confirm the role of serine and metallo-beta-lactamase enzymes in cefiderocol resistance.

**Conclusion:**

Cefiderocol resistance can be rapidly selected in *E. coli* through diverse mechanisms, including mutations affecting iron transport and cell wall biosynthesis, leading to significant phenotypic adaptations like the SCV phenotype. Continuous global surveillance is crucial to monitor the emergence of such resistance.

## 1 Introduction

Antibiotic resistance is one of the most serious public health challenges of the 21st century. The global health landscape is increasingly challenged by the rise of antibiotic resistance, which leads to elevated morbidity, mortality, and significant economic burdens (Wang et al., 2022; Kriz et al., 2024). Of particular concern are infections caused by multidrug-resistant (MDR) and extensively drug-resistant (XDR) Gram-negative bacteria (Almutairy, 2024; Elshamy and Aboshanab, 2020; Kriz et al., 2024; Kocer et al., 2023; Mubashar et al., 2025). Third generation cephalosporin-resistant and carbapenem resistant Enterobacterales [e.g., *Escherichia coli* (*E. coli*), *Klebsiella* spp.], carbapenem resistant *Acinetobacter baumannii* and MDR *Pseudomonas aeruginosa* are considered most problematic and hence identified as priority pathogens (Macesic et al., 2025).

*E. coli*, a medically significant pathogen responsible for urinary tract infections (UTIs), pneumonia, sepsis etc. is notably adept at acquiring resistance mechanisms, including those conferring carbapenem resistance, often via horizontal gene transfer of resistance genes (e.g., *bla*_NDM_, *bla*_KPC_, *bla*_OXA48_, *bla*_IMP_) (Karakonstantis et al., 2022; Kuo et al., 2016; Ma et al., 2022; Nadi et al., 2024).

The available arsenal of the most effective antibiotics is shrinking due to the increasing rate of antibiotic resistance to all useful antibiotics including third generation cephalosporins, carbapenems and colistin (Arafa et al., 2025). The escalating threat of antibiotic resistance underscores the critical need for novel therapeutic strategies and a deeper understanding of resistance mechanisms (Almutairy, 2024; Bui et al., 2024; Karakonstantis et al., 2022). Cefiderocol, a recently approved siderophore cephalosporin, represents a significant advancement in the field of drug discovery to cure antibiotic resistant infections caused by Gram negative bacteria. Its structure, like cefepime and ceftazidime, confers stability against many β-lactamases, while its catechol side chain enables siderophore-like activity, facilitating active transport across the outer membrane of Gram-negative bacteria via iron-uptake systems (Brauncajs et al., 2024; Karakonstantis et al., 2022; Parsels et al., 2021; Zhao et al., 2023). This unique mechanism, also called “Trojan Horse strategy” has resulted in potent activity of this class of antibiotics against a broad spectrum of MDR pathogens, including *E. coli*, *Pseudomonas aeruginosa*, *Acinetobacter baumanni* and *Klebsiella pneumoniae* in multicenter studies (Hackel et al., 2018; Karakonstantis et al., 2022; Simner et al., 2022).

Despite its efficacy against carbapenem-resistant isolates, reports of cefiderocol resistance have emerged globally (Jousset et al., 2023; Klein et al., 2022; Kocer et al., 2023; Kriz et al., 2024; Simner et al., 2022). Proposed mechanisms of cefiderocol resistance include production of certain carbapenemases and mutations affecting proteins involved in siderophore transport (Daoud et al., 2023; Moon et al., 2022; Teran et al., 2024; Yang et al., 2024). Furthermore, heteroresistance has been implicated in treatment failures and may serve as a precursor to stable resistance (Moon et al., 2022). However, the existing data is sometimes contradictory and remains insufficient for a comprehensive understanding of the resistance pathways in *E. coli.*

To address this knowledge gap, we induced cefiderocol resistance *in vitro* in clinical *E. coli* strains harboring at least one carbapenemase gene. By employing whole-genome sequencing (WGS) and transcriptomics analysis on the resistant mutants, this study aimed to elucidate the genetic and phenotypic adaptations underlying cefiderocol resistance in *E. coli*.

## 2 Materials and methods

### 2.1 Bacterial strains

This study utilized five *E. coli* strains: the reference strain ATCC 25922 and four clinical isolates. Each clinical strain was carbapenem-resistant, but initially susceptible to cefiderocol, and harbored a single distinct carbapenemase gene—namely, *bla*_IMP-4_ (strain K6606), *bla*_NDM-5_ (strain K66460), *bla*_KPC-2_ (strain K9197), and *bla*_OXA-48_ (strain K5812). All clinical isolates were obtained from different patients to ensure genetic diversity. Details of all bacterial strains used in the study are given in Supplementary Table S1.

### 2.2 *In vitro* induction of cefiderocol resistance

Resistance to cefiderocol was induced in the bacterial strains using a serial passaging method in iron-depleted cation-adjusted Mueller-Hinton broth (ID-CAMHB) with increasing concentrations of the antibiotic. Briefly, each strain was inoculated (1:1000 dilution from a 0.5 McFarland suspension) into a series of tubes containing cefiderocol at concentrations ranging from 0.03 to 128.0 μg/mL to a final volume of 2.5 mL. The cultures were incubated at 37 °C with shaking, and growth was assessed every 24 h by visual inspection of turbidity. Next, 2.5 μL inoculum from the ID-CAMHB tubes with the highest cefiderocol concentration showing visible growth was transferred daily (1:1000 dilution) into a fresh set of ID-CAMHB tubes with cefiderocol concentrations up to 128 μg/mL. This process was repeated until cefideocol resistant populations emerged. The tubes showing growth at ≥16 μg/mL were subjected to subculturing on the blood agar plates and incubated for 24–48 h at 37 °C. Next, 10 bacterial colonies were randomly picked, and pure culture was made on blood agar. Bacterial identity was confirmed using matrix-assisted laser desorption ionization-time of flight mass spectrometry (MALDI-TOF MS) to ensure purity of the bacterial culture. Minimum inhibitory concentration (MIC) was determined by broth microdilution (BMD) method. Clinical Laboratory Standards Institute, United States (CLSI) 2025 breakpoints for *Enterobacterales* were used in this study. Isolates exhibiting a MIC value ≥ 16 μg/mL were considered cefiderocol resistant. The cefiderocol resistant isolates were subsequently passaged in drug-free ID-CAMHB for seven consecutive days, with daily MIC monitoring (in triplicate), to confirm the stability of the resistant phenotype. Stable resistant mutants were stored at −80 °C for further analysis.

### 2.3 Antibiotic susceptibility testing

Antibiotic susceptibility profiling was performed using the VITEK 2 system (BioMérieux, United States). The MIC of cefiderocol was specifically determined in triplicate using the BMD method, in accordance with standard reference methods.

To investigate the contribution of specific β-lactamase types to resistance, the BMD method was also performed in the presence of the metallo-β-lactamase inhibitor dipicolinic acid (DPA) and the serine β-lactamase inhibitor avibactam (AVI). The antibiotic susceptibility profiles of all strains were assessed both prior to the induction of resistance and after the development of cefiderocol resistance.

### 2.4 Bacterial growth curve analysis

Growth kinetics were assessed by monitoring the optical density at 600 nm (OD_600_) over a 72-h period. An overnight bacterial culture was diluted to an initial OD_600_ of 0.09–0.10 in sterile Luria Bertani (LB) broth, followed by a further 100-fold dilution in the same medium. Aliquots (200 μL) of the bacterial suspension were dispensed into six replicate wells of a 96-well microplate. To prevent evaporation and contamination, each well was overlaid with 70 μL of sterile mineral oil. The OD_600_ was measured at 10-min intervals for 72 h using a digital microplate spectrophotometer (Multiskan, Thermo Scientific, United States).

### 2.5 Biofilm formation assay

Biofilm formation was assessed by measuring the biomass of adhered cells using crystal violet staining. Briefly, two to three bacterial colonies were inoculated into Mueller-Hinton Broth (MHB) supplemented with 2% sucrose and incubated at 37 °C for 24 h. The resulting culture was then adjusted to a turbidity of 0.5 McFarland standard in fresh MHB. A 200 μL aliquot of each bacterial suspension was transferred into six replicate wells of a sterile 96-well flat-bottom polystyrene microtiter plate. The plate was incubated statically at 37 °C for 48 h to allow biofilm development. After incubation, the planktonic cells and media were carefully aspirated, and the wells were gently washed twice with 200 μL of sterile saline to remove non-adherent cells. The adherent biofilms were fixed and stained with 150 μL of a 0.1% (w/v) crystal violet solution for 15 min at room temperature. Following staining, the dye solution was aspirated, and any unbound crystal violet was removed by washing the wells with phosphate-buffered saline (PBS). The plate was air-dried, and the bound dye was subsequently solubilized by adding 150 μL of 95% ethanol to each well. The optical density of the solubilized crystal violet was measured at 620 nm (OD₆₂₀) using a microplate spectrophotometer. The mean OD₆₂₀ value for each strain (wild-type and cefiderocol-resistant mutants) was calculated from the six replicate wells (Hassuna et al., 2024).

### 2.6 Transmission electron microscopy

Transmission electron microscopy (TEM) was employed to compare the cell envelope thickness between wild-type and cefiderocol-resistant *E. coli* strains. The sample preparation followed a standard protocol for biological specimens to achieve optimal ultrastructural preservation. Briefly, bacterial cells from a 20 mL overnight culture in LB broth were harvested by centrifugation at 8,000 rpm. The pellet was washed three times with phosphate-buffered saline (PBS) and subsequently fixed with 2.5% glutaraldehyde at 4 °C overnight. After fixation, the cells were rinsed with 0.1 M PBS and stored in the same buffer at 4 °C for an additional night. Post-fixation was then carried out using 1% osmium tetroxide in 0.1 M phosphate buffer (pH 7.4) for 1.5 h. Dehydration was performed through a graded series of acetone (50, 70, 90, and 100%) to remove all water content. The dehydrated samples were then infiltrated and embedded in Epon 812 resin, which was polymerized to form solid blocks. Ultrathin sections of approximately 70 nm in thickness were cut using an ultramicrotome. These sections were collected on grids and double-stained with uranyl acetate and lead citrate to further enhance contrast for cellular components. Imaging was performed using a JEM-1400 Plus transmission electron microscope (JEOL Ltd., Tokyo, Japan). The cell envelope thickness was measured of 30 randomly selected cells from each wild-type and resistant variant.

### 2.7 Whole genome sequencing, assembly and annotation

Whole Genome Sequencing (WGS) was carried out using Illumina HiSeq 2500 platform and nanopore sequencing on Flongle flow cells. Raw reads were filtered to remove low-quality sequences and adaptors using skewer and PoreChop,^1^ respectively. *De novo* assembly was conducted using SPAdes Genome Assembler v3.14.0 (Prjibelski et al., 2020) and Unicycler v0.4.8^2^ (Wick et al., 2017). Gene prediction was performed using Prokka 1.12^3^ (Seemann, 2014). The antimicrobial resistance genes and multilocus sequence types (MLST) were analyzed via the CGE server.^4^ The single nucleotide polymorphism (SNP) was called using Snippy^5^ with original strains as a reference. The genomic data is submitted to NCBI SRA (sequence read archive) database under the Bio Project No. PRJNA1315231.

### 2.8 Gene expression analysis

Gene expression analysis was performed using RNA sequencing (RNA-Seq). Bacterial isolates were cultured overnight in LB broth and then grown to mid-log phase in the presence of cefiderocol. RNA was extracted from four biological replicates for each bacterial variant following treatment. Sequencing libraries were prepared and sequenced on an Illumina platform. Strain K9197 and its resistance variant were selected as representatives for RNA-Seq analysis. Differential gene expression analysis was conducted by comparing the transcriptomic profiles of the resistant strains against the wild-type control under cefiderocol exposure, including carbapenemase gene *bla*_KPC-2_, and genes that may be related to iron transport, such as *acrA*, *acrB*, *acrD*, *baeR*, *baeS*, *btuB*, *cirA*, *envZ*, *fecA*, *fepA*, *fhuA*, *fhuE*, *fiu*, *ftsL*, *fyuA*, *iutA*, *mdtA*, *mdtB*, *mdtC*, *mexA*, *mexB*, *ompC*, *ompF*, and *ompR*. The RNA-Seq data has been submitted to The National Genomics Data Center (NGDC, Accession No. CRA042267).

### 2.9 Statistical analysis

Statistical analyses were performed using Minitab software version 19.1. Differences in biofilm formation between wild-type and cefiderocol-resistant strains were evaluated using Unpaired Student’s *T*-test. Similarly, the significance of differences in diameter measurements (e.g., inhibition zones or cell envelope thickness) was also assessed using Unpaired Student’s *t*-test. A *p*-value < 0.05 was considered statistically significant.

### 2.10 Ethics statement

The use of patient medical records and the *E. coli* isolates for research purposes was approved by the Ethics Committee of the China-Japan Friendship Hospital (2022-KY-133).

## 3 Results

### 3.1 *In vitro* development of cefiderocol resistance

**Figure 1:**
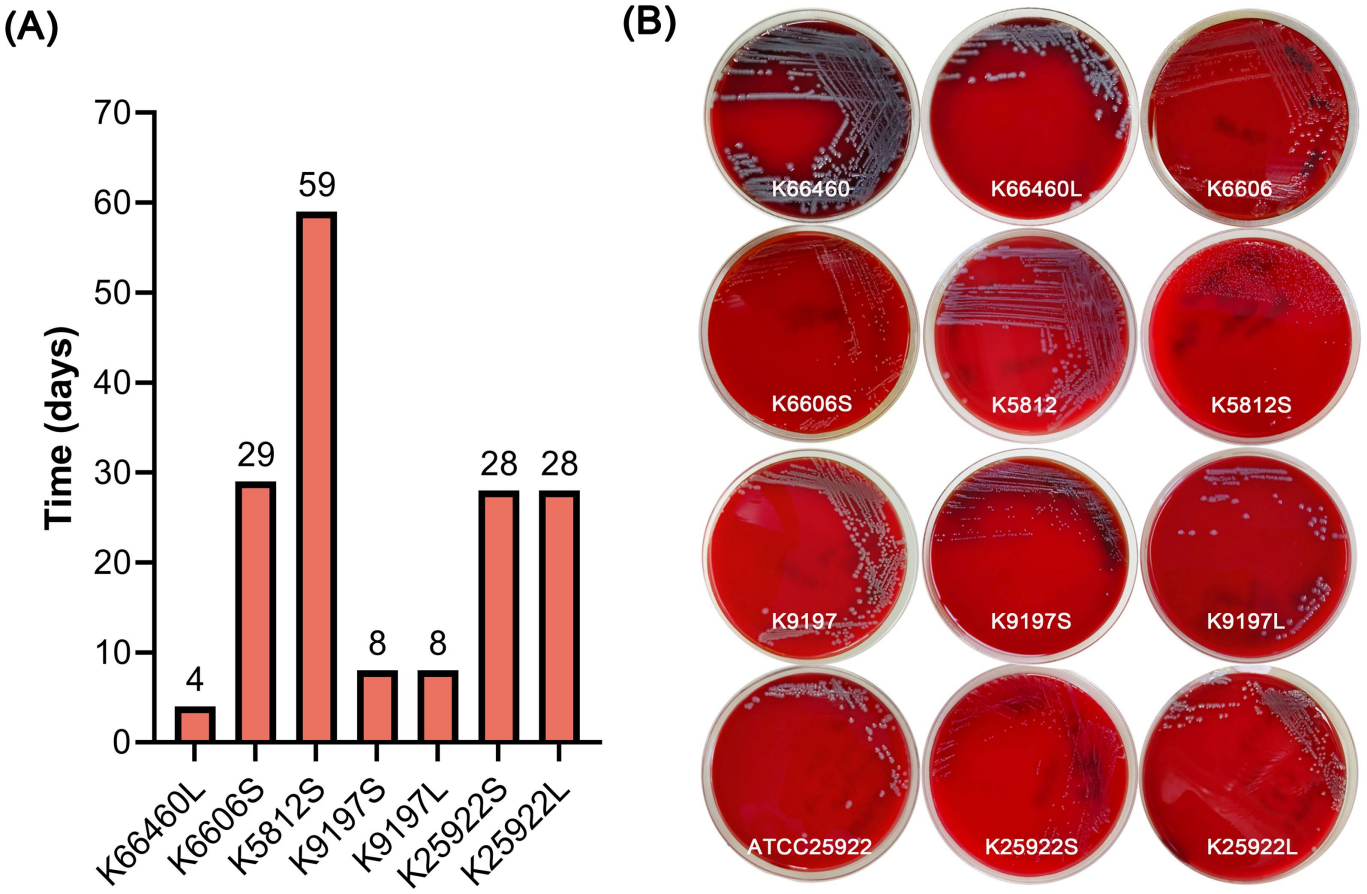
Time taken by each *E. coli* variant to acquire cefiderocol resistance **(A)** and colony morphology of original and FDC resistant variants **(B)**. No small colony variants emerged in the case of K66460.

Cefiderocol resistance was successfully induced in all clinical *E. coli* strains, as well as in the reference strain ATCC 25922, via serial passaging in increasing concentrations of the antibiotics. The time required for the development of resistance (defined as a MIC ≥ 16 μg/mL) varied among the strains, ranging from 4 to 59 days (Figure 1A). The resulting MICs for the resistant mutants ranged from 16 μg/mL to 128 μg/mL (Table 1).

**Table 1:** MIC values (μg/mL) to cefiderocol in the absence and presence of beta lactamase inhibitors.

Strain	Carbapenemase	FDC	FDC+DPA	FDC+AVI
K66460	*bla*_NDM-5_	0.5	0.125	–
K66460L	*bla*_NDM-5_	128	32	–
K6606	*bla*_IMP-4_	0.125	0.03	–
K6606S	*bla*_IMP-4_	16	2	–
K9197	*bla*_KPC-2_	1	–	0.06
K9197S	*bla*_KPC-2_	16	–	0.25
K9197L	*bla*_KPC-2_	16	–	0.25
K5812	*bla*_OXA-48_	0.25	–	0.03
K5812S	*bla*_OXA-48_	16	–	2
ATCC 25922	None	0.125	0.125	0.125
K25922S	None	16	16	16
K25922L	None	16	16	16

FDC, cefiderocol; DPA, dipicolinic acid; AVI, avibactam.

Notably, the kinetics of resistance development were influenced by the carbapenemase gene present. Strains harboring *bla*_NDM-5_ and *bla*_KPC-2_ developed resistance most rapidly. In contrast, the *bla*_OXA-48_-bearing strain (K5812) and the *bla*_IMP-4_-bearing strain (K6606) required a considerably longer duration to reach the resistance threshold.

During the induction process, we observed that growth in tubes with high drug concentrations did not always lead to stable resistance. Some populations exhibited a reversible tolerance phenotype, as their MICs returned to susceptible levels after subculturing in drug-free medium for 1–2 days. From the stable resistant populations, we selected 1 to 2 distinct colonies per strain for further analysis based on differences in MIC values, colony size, or morphology. The suffixes “S” (small) and “L” (large) were appended to the strain identification codes to denote the stable colony phenotype observed after the development of resistance. In the case of strain K9197, two stable morphotypes were recovered, namely K9197S and K9197L. For K9197L, the cefiderocol MIC was 8 μg/mL at the conventional 24–48 h reading time, but increased to 16 μg/mL after extended incubation (72–96 h); therefore, K9197L was classified as a cefiderocol-resistant derivative.

### 3.2 Impact of β-lactamase inhibitors on cefiderocol susceptibility

Consistent with the critical role of carbapenemases in cefiderocol resistance, susceptibility testing in the presence of β-lactamase inhibitors revealed significant MIC reductions. For wild-type and cefiderocol-resistant strains carrying carbapenemase genes, the addition of DPA (targeting metallo-β-lactamases) or AVI (targeting serine β-lactamases) reduced MIC values by 4- to 64-fold. In contrast, the MIC of ATCC 25922 (lacking β-lactamase genes) remained unchanged under inhibitor exposure.

The most pronounced effect was observed in the *bla*_KPC-2_-harboring strain K9197S, where AVI reduced the MIC from 16 μg/mL to 0.25 μg/mL (64-fold reduction). These results confirm that serine carbapenemases and metallo-β-lactamases contribute directly to cefiderocol resistance in both wild-type and induced-resistant strains (Table 1).

### 3.3 Emergence of small colony variants during cefiderocol exposure

Exposure to cefiderocol in liquid medium led to the emergence of small colony variants (SCVs) in four out of the five tested isolates (Figure 1B). This phenotypic shift was typically observed when the MIC values of the bacteria reached 2–4 μg/mL, not to resistance level. Initially, a mixture of small and large colonies was observed on blood agar plates; however, after 2–3 days, the entire population predominantly exhibited the SCV phenotype. The average normal bacterial colony had a diameter of 2–3 mm, while SCVs were measured ≤ 0.5 mm.

Colonies were carefully picked and streaked onto fresh blood agar to screen for stable SCVs. While many variants reverted to the normal colony morphology upon subculturing in drug-free liquid LB broth medium, a subset remained stably small even after seven consecutive passages in liquid LB broth without antibiotic pressure. Colony size of the SCVs on the blood agar was measured before and after stability subculturing in the liquid medium.

SCVs emerged in all strains except K66460L (carrying *bla*_NDM-5_), which developed resistance within only 4 days. To further assess SCV propensity in this strain, K66460L was cultured in ID-CAMHB containing cefiderocol (16 μg/mL) for 4 weeks, yet no SCVs were detected on blood agar.

Notably, some SCV isolates, such as K5812S, exhibited markedly delayed growth, requiring up to 2–3 days for visible colony formation. In these cases, BMD assays were extended to 72 h for accurate MIC determination. Additionally, a substantial proportion (55%, *n* = 55/100) of randomly selected SCVs of ATCC25922 (K25922S) could not be reliably identified by MALDI-TOF MS. We repeated the attempts several times by different people, with the same results. We picked several colonies (10–12 each time) of K25922S from the blood agar plates. In parallel, we tested colonies of other bacterial strains. Each time about half of the K25922S strains could not be identified. Repeated attempts (10X) by the same person or some other person could not helpful. Wild type-ATCC25922, K25922L (cefiderocol resistant strain, but not showing SCV phenotype), all other bacterial strains as well as SCVs of other *E. coli* strains (e.g., K9197S) were easily identified by MALDI TOF MS. We even sub-cultured an identified colony of K25922S and tried to identify few colonies by MALDI TOF MS from the blood plate on the fresh one. The same issue persisted each time, suggesting alterations in surface protein expression or sample preparation challenges associated with these SCV phenotypes.

### 3.4 Growth kinetics

**Figure 2:**
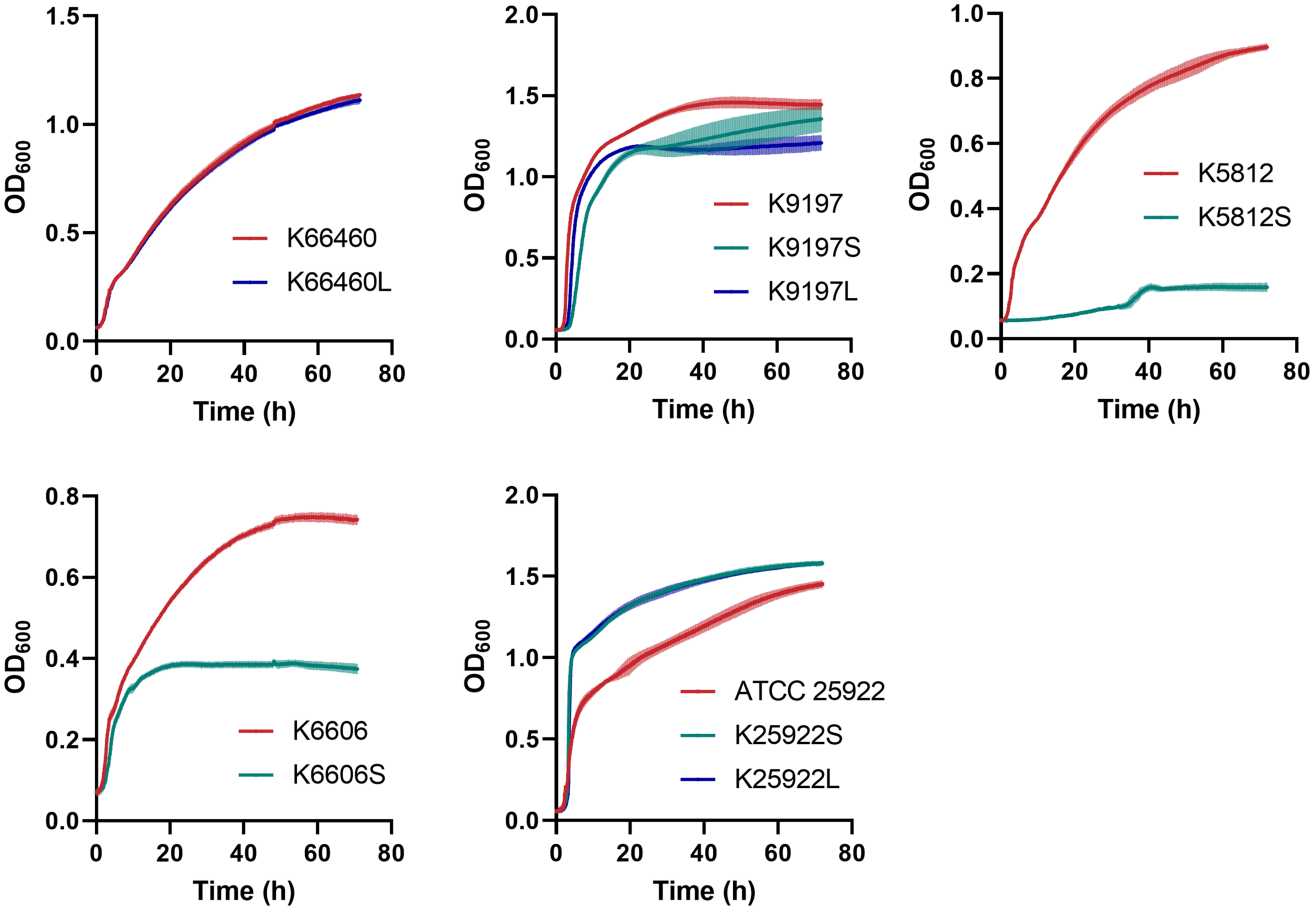
Optical density versus time curves of all original and cefiderocol resistant variants of *E. coli*. Significant difference in growth patterns has been observed in the case of original and small colony/drug resistant variants of K5812 and K6606. Longest lag phase (>24 h) and slowest growth rates were recorded for K5812S.

The growth kinetics analysis revealed significant differences between the wild-type strains and their cefiderocol-resistant variants, particularly those exhibiting the SCV phenotype (Figure 2). The resistant variant K5812S, which displayed a stable SCV morphology, demonstrated the most pronounced alteration in growth, characterized by an extended lag phase and the slowest overall growth rate among all tested isolates. In contrast, for strain K66460 and its resistant variant K66460L, which did not develop the SCV phenotype, no significant difference in growth kinetics was observed compared to the original susceptible isolate.

### 3.5 Biofilm production

Biofilm production varied among the cefiderocol-resistant mutants relative to their corresponding wild-type strains (Table 2). A slight decrease in biofilm formation was observed in three resistant variants, but these differences were not statistically significant. A statistically significant reduction in biofilm production (*p* < 0.0173) was observed in only one of the four resistant mutants tested. Therefore, although reduced biofilm formation was noted in some resistant derivatives, this effect was not consistent across all strains. We did not perform biofilm production assay for the SCVs of K5812S, which showed slowest growth rate both on solid as well as in the liquid medium (Figure 2). Visible colonies of this mutant strain appeared after 48–72 h, making the comparison with normal WT *E. coli* strain difficult using the same assay settings.

**Table 2:** A comparison of biofilm production by wild-type and cefiderocol resistant mutants.

Strain	OD_620_	S. D (±)	*p*-value
ATCC 25922	0.2772	0.3242	–
K25922S	0.035	0.0258	0.156311
K6606	0.0493	0.0213	–
K6606S	0.0196	0.0061	0.017314
K66460	0.0473	0.074	–
K66460L	0.0464	0.027	0.97839
K9197	0.0485	0.0378	–
K9197S	0.0335	0.0238	0.4738
K9197L	0.0195	0.0013	0.12463

### 3.6 Transmission electron microscopy analysis of cell envelope morphology

**Figure 3:**
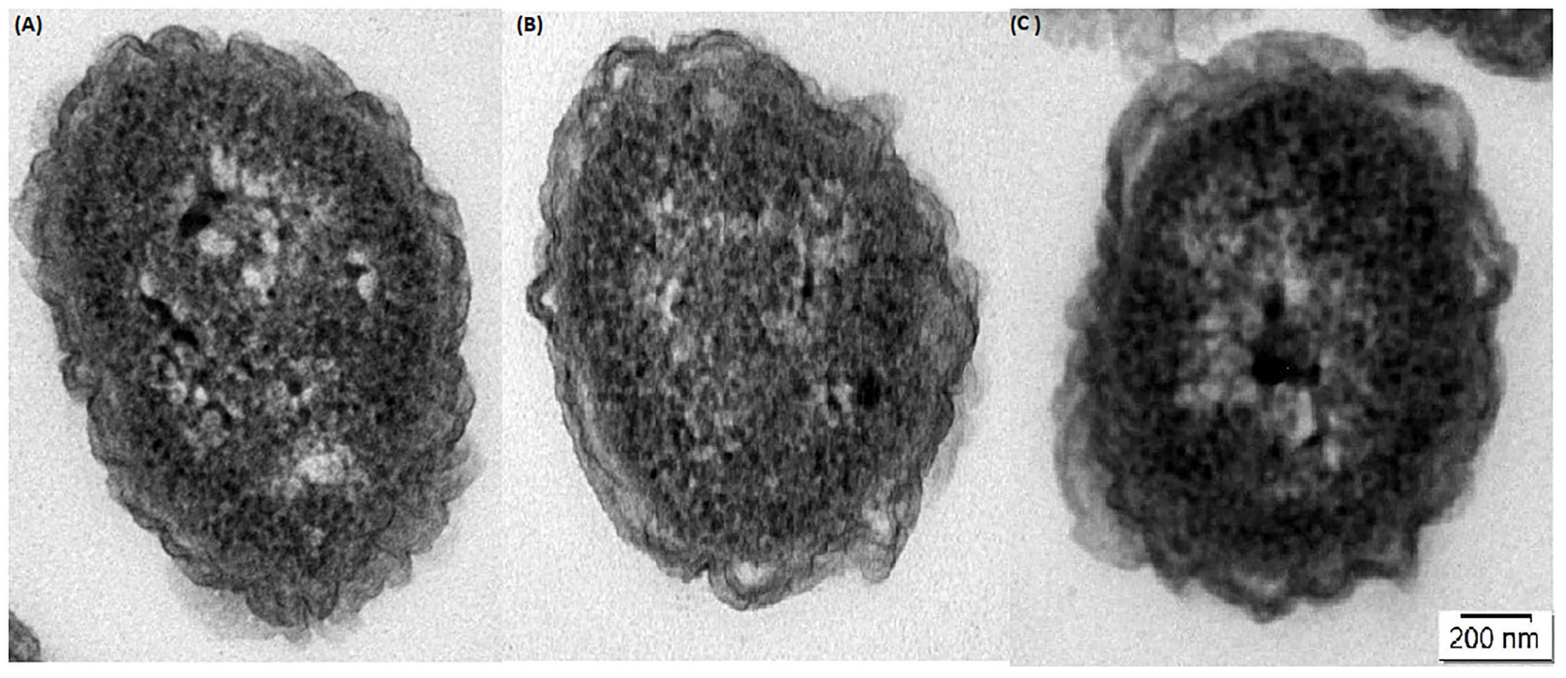
Transmission electron microscopy images of wild type ATCC25922 **(A)**, K25922S **(B)**, and K25922L **(C)** strains.

Transmission electron microscopy (TEM) revealed distinct alterations in cell envelope architecture between wild-type strains and cefiderocol-resistant variants. Notably, all small-colony variants (SCVs) exhibited thicker cell walls, and quantitative analysis demonstrated statistically significant difference (*p* = 2.1×10^−10^) in one of them (Table 3; Figure 3). In contrast, non-SCV cefiderocol-resistant mutants (e.g., K25922L) showed no thicker cell wall dimensions relative to parental strains.

**Table 3:** Measurements of cell wall diameters of cefiderocol susceptible strains and their resistant variants by TEM.

Strain	Cell wall diameter (nm)	S. D (±)	*p*-value
K6606	26.82	3.12	–
K6606S	38.92	8.05	0.00000000021
K5812	49.04	9.02	–
K5812S	53.60	11.03	0.084876682
ATCC25922	52.02	8.83	–
K25922S	55.12	6.577	0.128043229
K25922L	51.00	5.21	0.592113909

### 3.7 WGS analysis of cefiderocol resistance mechanisms

**Figure 4:**
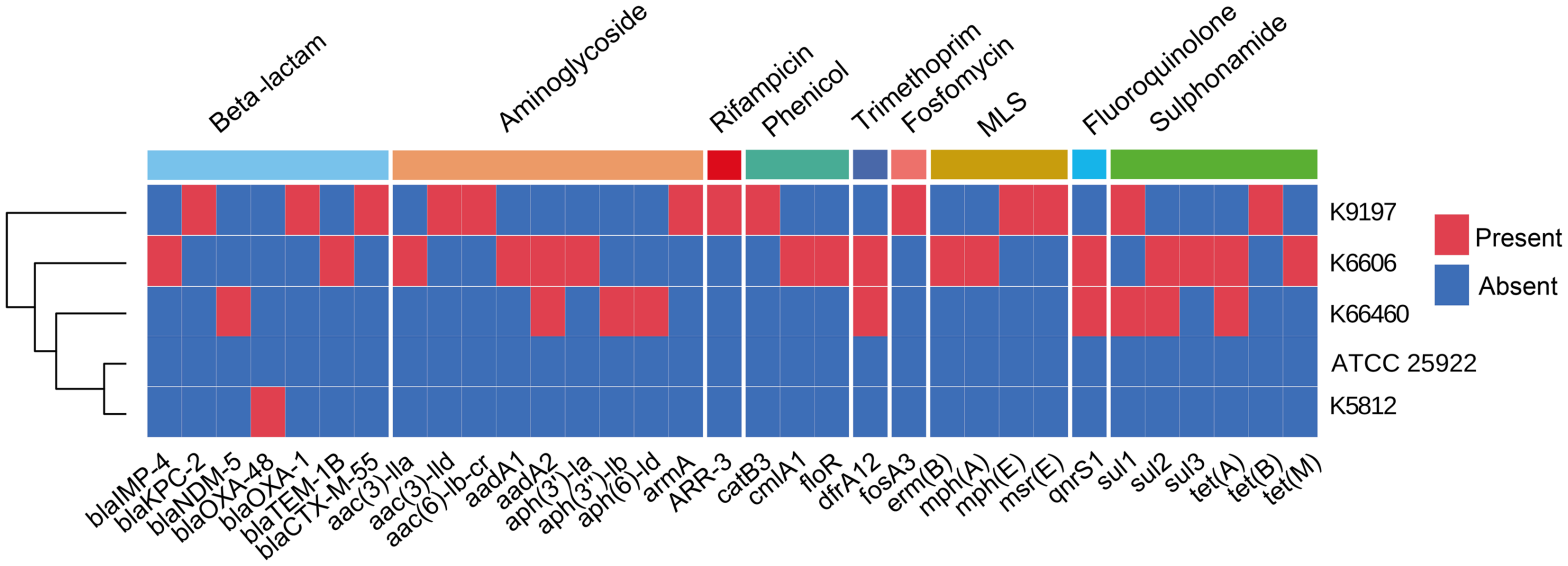
Antibiotic resistance genes of all the original *E. coli* strains.

The four clinical *E. coli* strains K5812, K66460, K6606, and K9197 were classified as ST69, ST4538, ST457, and ST405, respectively. Only one copy of the carbapenemase gene was present in all wild-type and cefiderocol-resistant strains (Details of the resistance genes are presented in Figure 4). Comparative genomic analysis identified 16 non-synonymous mutations across resistant lineages (Table 4). Among these, several genes (e.g., *envZ*, *tonB*, *fiuA*, and *cirA*) have previously been implicated in cefiderocol resistance and therefore represent plausible candidate determinants of the cefiderocol-resistant phenotype observed in our strains. Notably, mutation in *envZ* gene occurred most frequently (4/7 resistant variants). Mutations in other genes may also represent novel candidates for cefiderocol resistance.

**Table 4:** Detail of all mutations occurred in cefiderocol resistant variants analyzed by WGS.

Strain	Mutation	DP value	AF value	Gene	Function/protein encoded	Amino acid
K66460L	1341-1347Del	155	1.0	*cirA*	Colicin I receptor	Truncated
	T424C	90.0	1.0	*cobA*	Precorrin 2 oxidase	P133L
	G1553A	159	1.0	*fiuA*	Ferrichrome-iron receptor	G518E
	T424C	90	1.0	*tssI*	vgrG	Silent
K6606S	C549A	183	1.0	*envZ*	Sensory histidine kinase protein	N183K
	A114C	161	1.0	*ybbW*	Allantoin permease	Silent
	G116T	138	1.0	*aspS*	Aspartyl-t RNA synthetase	R39L
	571InsTTA	849	0.996	*bla*_IMP-4_	Subclass B1 metallo beta lactamase IMP-4	INS191L
K5812S	C126G	147	1.0	*envZ*	Sensory histidine kinase protein	S42R
	G1271A	313	0.99	*valS*	Valyl-tRNA synthetase	C424Y
K9197S	G219T	183	1.0	*ubiB*	Ubiquitin biosynthesis regulatory protein kinase	H73Q
	T72C	73	1.0	*insA*	IS1A family transposase	Silent
K9197L	T72C	72	1.0	*insA*	IS1A family transposase	Silent
	G218A	156	1.0	*lipA*	Lipoyl synthase	C73Y
	T3917C	167	1.0	*irp2*	Iron aquisition yersiniabactin synthesis enzyme	L1306S
K25922S	T722G	160	1.0	*envZ*	Sensory histidine kinase protein	V241G
	G309T	209	1.0	*slyD*	FKBP-type peptidyl-prolyl cis-trans isomerase SlyD	Silent
	T596C	166	1.0	*baeS*	Sensory Histidine Kinase	L199P
K25922L	T596C	170	1.0	*baeS*	Sensory Histidine Kinase	L199P
	208-213Del	119	1.0	*tonB*	TonB-ExbBD energy transducing system	E70-P71Del
	T722G	171	1.0	*envZ*	Sensory histidine kinase protein	V241G
	G309T	184	1.0	*slyD*	FKBP-type peptidyl-prolyl cis-trans isomerase SlyD	Silent

Our data did not identify any previously characterized SCV-associated mutation pattern in these isolates. This observation suggests that SCV formation in our study may involve complex and potentially multifactorial processes. Some of the mutations listed in Table 4 may represent candidate genetic correlates of the SCV phenotype; however, these associations are observational and require functional validation. A *bla*_IMP-4_ deletion was uniquely identified in resistant variant K6606S (Table 4), representing a previously unreported genetic adaptation.

### 3.8 Transcriptomic profiling of K9197S reveals strain-specific alterations in iron transport and membrane-associated functions

RNA sequencing analysis of the cefiderocol-resistant strain K9197S identified significant transcriptomic reprogramming relative to its wild-type parent strain, with 1,362 differentially expressed genes (DEGs). Among these, 554 genes were up-regulated and 808 were down-regulated (Supplementary Figure S1). Notably, expression of the carbapenemase gene *bla*_KPC-2_ remained unchanged between wild-type strain K9197 and its cefiderocol-resistant variant K9197S, indicating that this resistance determinant was not transcriptionally modulated in this lineage under cefiderocol pressure. Because RNA-Seq was performed only for K9197S, these findings should be interpreted as strain-specific and not as universally representative of all cefiderocol-resistant isolates examined in this study.

Among the 1,362 DEGs, we prioritized genes with the most direct mechanistic relevance to cefiderocol uptake and envelope permeability. Comparative analysis identified three such genes involved in membrane transport and iron acquisition that were significantly down-regulated in K9197S (fold change > 2; adjusted *p* ≤ 0.05): the outer membrane porin gene *ompF*, the yersiniabactin receptor gene *fyuA*, and the ferric citrate transporter gene *fecA*. These genes were highlighted because they represent biologically plausible candidates linking the transcriptomic dataset to known cefiderocol entry pathways. Their coordinated suppression in K9197S suggests a potential adaptive mechanism whereby reduced membrane permeability and altered iron homeostasis may contribute to cefiderocol resistance in this mutant.

## 4 Discussion

Cefiderocol has emerged as a promising therapeutic option for infections caused by multidrug-resistant (MDR) Gram-negative bacteria (Karakonstantis et al., 2022; Simner et al., 2022; Zhao et al., 2023). However, resistance cases are increasingly being reported worldwide. In this study, we successfully induced cefiderocol resistance in four clinical isolates and one control strain of *E. coli* using *in vitro* serial passaging. Our approach aligns with previous attempts to generate resistance *in vitro* by exposing bacteria to sublethal drug concentrations, which have commonly reported mutations in iron transport proteins (e.g., the siderophore receptors CirA and FiuA), beta-lactamase production (such as NDM-5 and KPC-2), and/or alterations in outer membrane proteins (Kriz et al., 2024; Lu et al., 2025; Simner et al., 2022).

Building on this established approach, we identified several genetic and transcriptional alterations associated with cefiderocol resistance in *E. coli*. Notably, a mutation in the sensory histidine kinase gene *envZ* was detected in four out of seven cefiderocol-resistant mutants analyzed by WGS. Mutations in this gene have been previously implicated in resistance by other research groups. The EnvZ-OmpR two-component system regulates the expression of outer membrane porins OmpF and OmpC in response to osmolarity changes (Gerken et al., 2024; Wang et al., 2022), and mutations in *envZ* can lead to reduced porin expression, thereby decreasing bacterial permeability and potentially lowering susceptibility to antibiotics, including cefiderocol (Gerken et al., 2024; Pérez-Palacios et al., 2024).

These genomic observations are complemented by transcriptomic analysis of the representative resistant mutant K9197S, which revealed expression changes that may be relevant to resistance in this specific genetic background. Among the 1,362 DEGs, we focused on *ompF*, *fyuA*, and *fecA* because these genes are directly related to outer membrane permeability and iron uptake, two processes with clear mechanistic relevance to cefiderocol entry. The significant downregulation of these genes in K9197S indicates a coordinated response in this mutant to limit membrane permeability and alter iron homeostasis. As cefiderocol relies on iron transport systems for cellular entry, suppression of these transporters in K9197S represents a plausible adaptive strategy. The unchanged expression of *bla*_KPC-2_ in resistant variants suggests that transcriptional regulation of this carbapenemase gene does not contribute significantly to cefiderocol resistance in our model.

Given that resistance may involve multiple mechanisms, such as beta-lactamase activity, altered iron transport, and sensory kinase mutations, we specifically analyzed resistant mutants of ATCC 25922, which lacks acquired beta-lactamase genes. Both its SCV (K25922S) and normal-sized (K25922L) mutants took longer to develop resistance compared to *bla*_NDM-5_ or *bla*_KPC-2_-producing strains. Resistance in these mutants was not attributable to a single gene; both carried missense mutations in two sensory histidine kinase genes (*envZ* and *baeS*), and K25922L also harbored a frameshift mutation in *tonB*.

Susceptibility testing in the presence of beta-lactamase inhibitors further clarified the role of different mechanisms. A significant reduction in MIC was observed for both susceptible and resistant strains carrying beta-lactamases when grown with inhibitors, whereas no change was seen in ATCC25922-derived mutants. This confirms the contribution of serine and metallo-beta-lactamases to cefiderocol resistance in those strains. The effect was most pronounced (up to 64-fold reduction) in K9197 and its mutants (K9197S and K9197L). The role of *bla*_NDM-5_ and *bla*_KPC-2_ in cefiderocol resistance has been documented in several studies across different bacterial species, including *E. coli* (Hong et al., 2024; Nurjadi et al., 2022; Sadek et al., 2025; Wang et al., 2022), and our results are consistent with these reports.

Previous studies have reported no or limited role of *bla*_OXA-48_ in cefiderocol resistance (Fröhlich et al., 2022; Malisova et al., 2023). However, recent evidence suggests that *bla*_OXA-48_ can contribute to resistance in certain contexts (Baltas et al., 2024). In our study, 8-fold decrease in MIC was observed when the wild-type *bla*_OXA-48_-harboring strain and its mutants were grown with beta-lactamase inhibitors. Little was known about the role of *bla*_IMP_ genes in cefiderocol resistance. Here, the *bla*_IMP-4_-carrying strain K6606 developed resistance, and a 4-fold decrease in MIC occurred in the presence of a beta-lactamase inhibitor, providing novel evidence for *bla*_IMP-4_’s involvement in cefiderocol resistance.

In addition, a unique and significant aspect of our study is the frequent emergence of SCVs following cefiderocol exposure. SCVs represent a serious clinical challenge that is often underreported or missed in routine diagnostics (Gil-Gil et al., 2024). In our work, SCVs emerged in three clinical strains (carrying *bla*_KPC-2_, *bla*_OXA-48_, and *bla*_IMP-4_, respectively) and the control strain ATCC 25922 (which lacks a carbapenemase gene) within 2 weeks of cefiderocol exposure. The cefiderocol resistance developed in these strains was stable, as it persisted after seven consecutive days of subculturing in drug-free medium. Interestingly, no SCVs emerged from the *bla*_NDM-5_-carrying strain K66460, even though it developed resistance rapidly (within 4 days) and was further incubated under drug pressure for 4 weeks. One of the SCV-producing strains, K5812S exhibited the slowest growth rates with visible colonies appearing only after 48–72 h. Although most SCVs in our study reverted to a normal phenotype after growth in drug-free medium, we successfully isolated and characterized several stable SCV lineages. Additionally, we encountered difficulties in identifying some SCVs using MALDI-TOF MS, suggesting alterations in their protein expression profiles.

Much of the existing SCV research has focused on *Staphylococcus aureus*, with limited data available for *E. coli* and other Gram-negative pathogens. The SCVs phenomenon has been a known contributor in causing recurrent and persistent infections that may also be associated with antibiotic resistance. Emergence of SCVs pose both diagnostic and clinical challenges to health care professionals, since the phenomenon may translate into serious consequences and may challenge the existing practices, especially in the poor resource settings. Majority of diagnostic laboratories still rely upon manual methods of bacterial identification, largely upon microscopy, cultural characteristics and biochemical tests. The results of our study reveal that, apart from smaller colony size, SCVs may take up to 3 days to develop a visible bacterial colony, which may lead to misidentification and underreporting of bacteria. On one hand existing data related to SCVs is limited to just a few bacterial species, on the other hand very limited number of antibiotics are reported to induce formation of SCVs.

Studies on *S. aureus* SCVs indicate diverse molecular mechanisms, often involving mutations in genes such as *hemB* and *lipA* (Becker, 2023; Noaman et al., 2023). In contrast, WGS analysis of our stable SCVs did not reveal mutations in these previously reported genes. Instead, we identified several novel mutations (Table 4), including SNPs in genes encoding aspartyl-tRNA synthetase and valyl-tRNA synthetase (involved in protein synthesis), an A > C SNP in *ubiB* (required for ubiquinone biosynthesis and respiratory electron transport) (Poon et al., 2000), and a large deletion in *yabI* (encoding an inner membrane protein of the DedA family) (Kumar and Doerrler, 2014). These findings suggest that SCV formation in *E. coli* under cefiderocol pressure may involve distinct genetic pathways affecting core metabolism and cell envelope integrity. Mutations in the genes involved in amino acid synthesis as well as other core pathways suggest their potential role in impaired growth and bacterial response in the form of emergence of SCVs, rather than confirmed causal determinants. Nevertheless, the relationship between individual mutations and the observed SCV phenotype remains correlative in the present study and will require functional validation through gene knockout, allelic replacement, complementation, and/or gene editing experiments.

In addition to genetic basis, the mechanism of SCVs development also seems to be regulated by differential gene expression at transcription and translation level. Since present study was largely focused on phenotypic and genetic aspect, multi-omics approaches such as transcriptomics, proteomics and metabolomics may further explain the mechanism.

Growth curve analysis revealed significant differences between wild-type and resistant SCV mutants of K6606 and K5812. Resistant mutants in both cases showed lower maximum optical density, with the longest lag phase and slowest growth rates observed in K5812S. This SCV behavior, atypical for the normally fast-growing *E. coli*, poses a serious diagnostic and clinical challenge, potentially leading to underestimation in routine lab cultures. Our difficulty in identifying some SCVs by MALDI-TOF MS further indicates substantial physiological alterations.

This correlation underscores that the observed growth impairment is specifically associated with the SCV phenotype in cefiderocol-resistant mutants. The reduced growth rate and metabolic adaptations are consistent with typical SCV physiology, which often involves compromised metabolic efficiency, and difficulty in treating infections. SCV formation has previously been reported in *E. coli* by Noaman et al. (2023) by treating with gentamycin. However, a comparison of cell walls has been made for the first time in our study (to the best of our knowledge). Correspondingly, transmission electron microscopy revealed that SCVs frequently possessed thicker cell walls compared to their wild-type counterparts and non-SCV resistant mutants, suggesting that cell wall remodeling is part of the SCV adaptation induced by cefiderocol exposure, potentially contributing to both the observed growth defects and antibiotic tolerance.

A previous study has reported anti-biofilm activity of cefiderocol, in combination with imipenem (Ferretti et al., 2024). However, limited knowledge was available on production of biofilm by bacterial cells resistant to cefiderocol. Since cefiderocol resistance involves mutation in several important metabolic pathways [e.g., those involved in iron transport through siderophore production or some other pathways (also causing SCVs formation)], investigation of biofilm production by these bacteria was of profound interest. In the present work, wild-type *E. coli* strains and their resistant mutants were tested and compared for their capability to produce biofilms using *in vitro* experiment. Our results reveal that there was reduction in biofilm production in cefiderocol resistant strains (both normal and SCVs). However, the magnitude of biofilm production and difference between the wild-type and mutant strains differed. Significant difference (*p* ≤ 0.05) was observed between K6606 and its resistant mutant. Our results agree with a study conducted by Bao et al. (2022), which reports reduced expression of genes involved in biofilm synthesis by proteomics and transcriptomics analysis. In our study, some of the SCVs (e.g., K5812S) showed unusually slower growth rate, producing visible colonies after 2–3 days, a comparison of biofilm production could not be made with WT strain (Figure 2).

Taking together, SCVs formation during treatment may complicate disease management and pose risk of formation and spread of SCVs. The existence of SCVs is not rare, but difficult to recover from clinical samples, as mentioned above (Proctor et al., 2006). SCVs have been recovered from both chronic as well as aggressive infections. Furthermore, antibiotics induced SCVs development is not a new phenomenon. Several other antibiotics have also been reported to play a role in induction of SCVs formation. A combination of both cefiderocol resistance and SCVs is a clinical challenge (Noaman et al., 2023; Santos and Hirshfield, 2016). Since there is very limited data available on immune response, antibiotic resistance, physiology and virulence of SCVs of *E. coli* as well as other Gram-negative bacteria, there is a need of future focused *in vivo* studies. The results of our study underscore the need for further studies to be conducted by different hospitals for continued surveillance and monitoring of emergence and dissemination of SCVs in health-care settings.

This study has several limitations. The *in vitro* induction model may not fully replicate the host environment, and the functional consequences of identified mutations require validation through genetic experiments. In addition, transcriptomic analysis was performed on only one cefiderocol-resistant mutant (K9197S); therefore, these RNA-seq findings should be considered exploratory and strain-specific, and additional transcriptomic analyses of independent resistant lineages will be required to determine whether the observed expression changes are broadly shared. Furthermore, experimental evidence of role of each mutation in SCVs needs to be determined by gene knock out and/or gene editing. Future work may investigate the stability of these resistance mechanisms *in vivo* and explore combination therapies that might prevent SCV emergence. We also aim to further investigate the role of each of these mutations in SCVs development. In addition, our future project will involve further unraveling the mechanism of SCV formation in these strains using multi-omics approach including transcriptomics, metabolomics and proteomics.

In conclusion, our integrated genomic, transcriptomic, and phenotypic analysis reveals that *E. coli* employs diverse strategies to overcome cefiderocol pressure, including target enzyme production, membrane permeability reduction, iron transport alteration, and SCV formation. However, the transcriptomic findings are based on a single resistant mutant and should therefore be interpreted as hypothesis-generating rather than universally representative.

The transcriptomic findings are based on a single resistant mutant, and the observed associations between specific mutations and the SCV phenotype remain hypothesis-generating pending functional validation.

These findings underscore the need for comprehensive resistance monitoring and the development of novel approaches to counter these adaptive mechanisms.

## Data Availability

The datasets presented in this study can be found in online repositories.The names of the repository/repositories and accession number(s) can be found in the article/Supplementary material.

## Footnotes

1
https://github.com/rrwick/Porechop

2
https://github.com/rrwick/Unicycler

3
https://github.com/tseemann/prokka

4
https://cge.cbs.dtu.dk/services/

5
https://github.com/tseemann/snippy

## Supplementary material

Supplementary Figure S1 A comparison of gene expressions in K9197S and K9197.
