# Supplementary figures and images for: Small colony variants and cefiderocol resistance in clinical Escherichia coli: an in vitro mechanistic study
Source: Front Microbiol. 2026 May 26;17:1761368. doi: 10.3389/fmicb.2026.1761368 (PMC13246609; doi:10.3389/fmicb.2026.1761368)

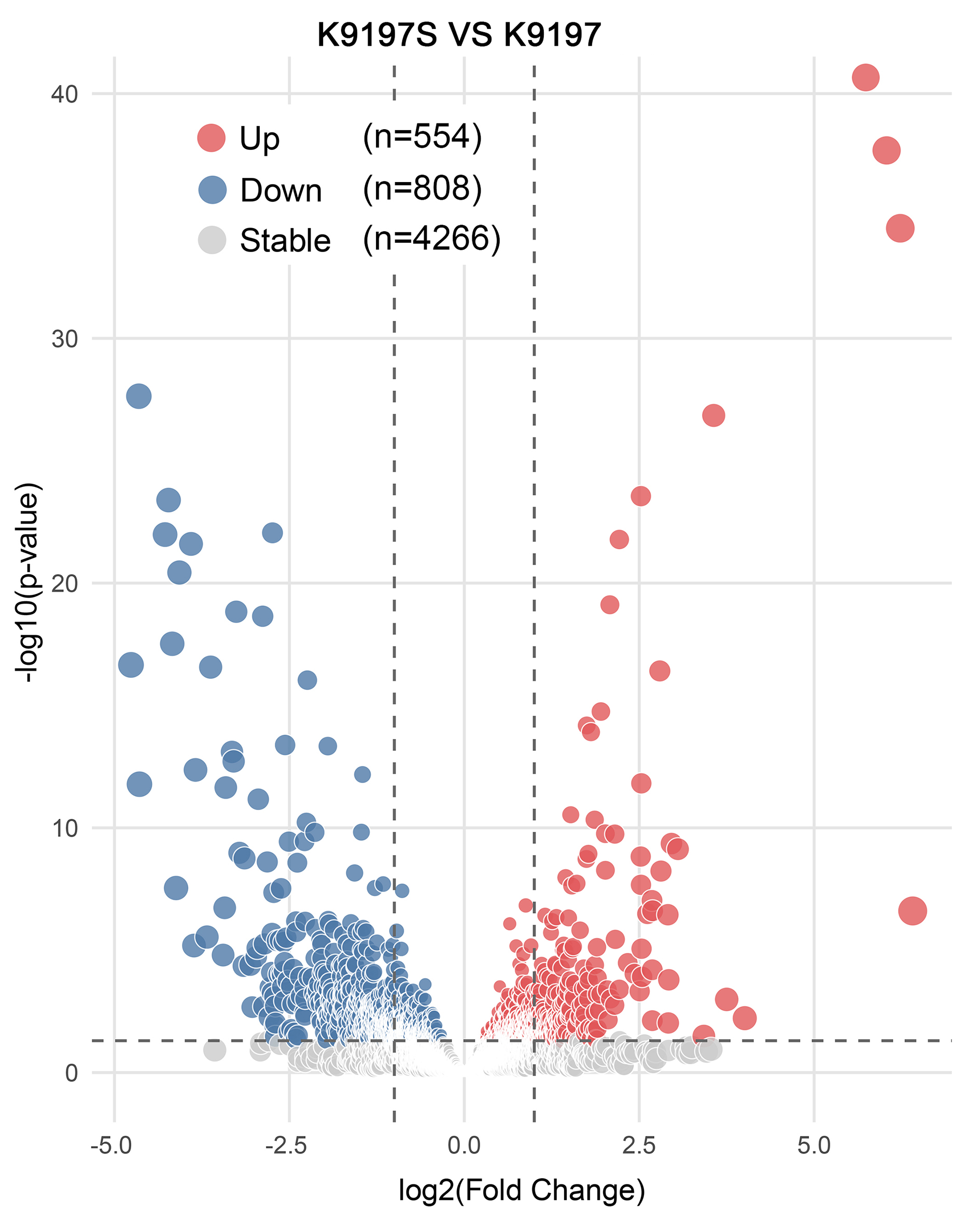

Supplement: Supplementary Figure S1 — A comparison of gene expressions in K9197S and K9197. [file Image_1.TIF]
