# Supplementary material for: Small colony variants and cefiderocol resistance in clinical Escherichia coli: an in vitro mechanistic study
Source: Front Microbiol. 2026 May 26;17:1761368. doi: 10.3389/fmicb.2026.1761368 (PMC13246609; doi:10.3389/fmicb.2026.1761368)
Supplement: Supplementary file 2 [file Table_1.DOCX]

**Table S1: Detail of the bacterial isolates used in this study**

| Strain I.D | Year of collection | Country | City | Infection type | Sample type |
| --- | --- | --- | --- | --- | --- |
| K6606 | 2019 | China | Shandong | Unkonwn | Sputum |
| K5812 | 2018 | China | Shanghai | Unkonwn | Unkonwn |
| K9197 | 2022 | China | Beijing | Unkonwn | Sputum |
| K66460 | 2023 | China | Beijing | Unkonwn | Blood |
| ATCC25922 | - | - | - | - | - |
