# Supplementary material for: Small colony variants and cefiderocol resistance in clinical Escherichia coli: an in vitro mechanistic study
Source: Front Microbiol. 2026 May 26;17:1761368. doi: 10.3389/fmicb.2026.1761368 (PMC13246609; doi:10.3389/fmicb.2026.1761368)
Supplement: Supplementary file 3 [file Table_2.DOCX]

**Table S2. Detailed information of the WGS data used in the study.**

| **S. No.** | **Strain I.D.** | **Genome size (bp)** | **No. Of Contigs** | **G+C Content (%)** | **N50** | **N90** | **Coverage** | **Sequence Type** | **NCBI Accession:** | **NCBI**  **Bioproject No.** |
| --- | --- | --- | --- | --- | --- | --- | --- | --- | --- | --- |
| 1 | K66460 | 4,997,755 | 132 | 50.6 | 201659 | 9 | 217X | 4538 | SRR35257692 | PRJNA1315231 |
| 2 | K66460L | 5,007,303 | 136 | 50.6 | 168115 | 11 | 174.3X | 4538 | SRR35257691 | PRJNA1315231 |
| 3 | K6606 | 5,090,769 | 138 | 50.5 | 144951 | 12 | 220.8X | 457 | SRR35257690 | PRJNA1315231 |
| 4 | K6606S | 5,055,748 | 143 | 50.5 | 137876 | 12 | 233.6X | 457 | SRR35257689 | PRJNA1315231 |
| 5 | K5812 | 5,034,903 | 101 | 50.6 | 295119 | 7 | 209.3X | 69 | SRR35257700 | PRJNA1315231 |
| 6 | K5812S | 5,037,267 | 121 | 50.7 | 230736 | 8 | 199.2X | 69 | SRR35257699 | PRJNA1315231 |
| 7 | K9197 | 5,288,654 | 184 | 50.6 | 131562 | 12 | 210.0X | 405 | SRR35257688 | PRJNA1315231 |
| 8 | K9197S | 5,286,687 | 156 | 50.6 | 131562 | 12 | 214.3X | 405 | SRR35257687 | PRJNA1315231 |
| 9 | K9197L | 5,291,098 | 191 | 50.6 | 131562 | 12 | 208.1X | 405 | SRR35257698 | PRJNA1315231 |
| 10 | ATCC25922 | 5,157,445 | 136 | 50.4 | 205409 | 7 | 202.8X | 73 | SRR35257696 | PRJNA1315231 |
| 11 | K25922S | 5,107,758 | 131 | 50.5 | 205409 | 7 | 225.2X | 73 | SRR35257696 | PRJNA1315231 |
| 12 | K25922L | 5,107,605 | 125 | 50.5 | 205409 | 7 | 210.5x | 73 | SRR35257695 | PRJNA1315231 |
